# Human Fall Detection Using 3D Multi-Stream Convolutional Neural Networks with Fusion

**DOI:** 10.3390/diagnostics12123060

**Published:** 2022-12-06

**Authors:** Thamer Alanazi, Ghulam Muhammad

**Affiliations:** Department of Computer Engineering, College of Computer and Information Sciences, King Saud University, Riyadh 11543, Saudi Arabia

**Keywords:** human fall detection, deep learning, convolution neural networks, fusion networks, 3D-CNN and 2D-CNN

## Abstract

Human falls, especially for elderly people, can cause serious injuries that might lead to permanent disability. Approximately 20–30% of the aged people in the United States who experienced fall accidents suffer from head trauma, injuries, or bruises. Fall detection is becoming an important public healthcare problem. Timely and accurate fall incident detection could enable the instant delivery of medical services to the injured. New advances in vision-based technologies, including deep learning, have shown significant results in action recognition, where some focus on the detection of fall actions. In this paper, we propose an automatic human fall detection system using multi-stream convolutional neural networks with fusion. The system is based on a multi-level image-fusion approach of every 16 frames of an input video to highlight movement differences within this range. This results of four consecutive preprocessed images are fed to a new proposed and efficient lightweight multi-stream CNN model that is based on a four-branch architecture (4S-3DCNN) that classifies whether there is an incident of a human fall. The evaluation included the use of more than 6392 generated sequences from the Le2i fall detection dataset, which is a publicly available fall video dataset. The proposed method, using three-fold cross-validation to validate generalization and susceptibility to overfitting, achieved a 99.03%, 99.00%, 99.68%, and 99.00% accuracy, sensitivity, specificity, and precision, respectively. The experimental results prove that the proposed model outperforms state-of-the-art models, including GoogleNet, SqueezeNet, ResNet18, and DarkNet19, for fall incident detection.

## 1. Introduction

When older adults fall, there is a potential for severe injury or even death if the victim does not receive help immediately. A human fall is an unintended event involving sequences of sitting or standing, falling from a higher to a lower level, and being at rest. Several studies have concluded that falls are the leading reason for trauma among individuals with special needs, such as seniors. This is a reality acknowledged by the World Health Organization, cited in [1], which reports that 30 percent of the population older than 65 experiences one trauma incident a year that can be attributed to falls. Added to this, 47 percent of adults that fall lose their independence [2]. The above statistics indicate a need for immediate assistance when older adults fall. This need has spurred intensive activities in technologies involving fall detection. These activities are being encouraged by the rapid growth in video monitoring and communication technologies.

In the last few years, the proliferation of attention on fall detection has resulted in the development of numerous fall detection approaches [3,4]. Some techniques use information collected by devices such as acceleration and vibration sensors [3,4]. To detect falls, such methods may use human movement, sound, and vibration [4,5,6]. Nonetheless, these methods generally tend to have limitations in terms of performance. For example, noise can affect the acoustic sensors. Moreover, the use of floor vibration sensors is only possible if surfaces have sensors. It is also important to consider the costs involved in installing sensors in a large area, making some of the methods not a practical solution. Many people looking for more practical solutions are embracing common devices such as smartwatches and smartphones.

Other standard models in fall detection include focusing on data collected from video sequences [7]. These approaches can use a single camera, multiple cameras [8], omnidirectional ones [9], and stereo-pair cameras [10]. Undoubtedly, a camera can provide more details regarding a monitored person’s motion, and tries to extract crucial features from the video frequency so that it can determine if there has been a fall. This implies that the information gathered by a camera is more informative and richer when compared to that collected by ordinary sensors. Specifically, fall detection based on vision not only has the potential to save lives, but could also lower medical expenses related to accidents associated with fatal falls, particularly among older adults.

Previous studies have often divided human fall detection into two methods. The first involves the use of wearable sensors. Even though these have a high accuracy and tend to be cheaper, their limitation is that they are extremely intrusive. The second technique involves computer vision, including machine and deep learning methods. These have a high degree of accuracy, are highly robust, and are less intrusive.

The field of computer vision deals with the way a computer can comprehend information extracted from videos or digital images [11]. Probability, statistics, geometry, and learning theory are crucial in constructing computer vision models. Through employing videos and images from digital cameras with the assistance of deep learning models, it is possible to make machines identify and classify objects and make them react based on what they “see”.

Under the broad category of deep learning, there is a huge amount of techniques incorporating hierarchical probabilistic models, neural networks, and an array of supervised and unsupervised feature learning algorithms. The performance of deep learning methods is better when compared to previous state-of-the-art machine learning techniques in several fields [12,13]. Deep learning consists of models with multiple layers. These models can learn from data at numerous abstraction levels. This is the reason behind its ability to comprehend large and complicated amounts of data and use them to provide helpful information. There are numerous models that exist in the field of deep learning, including long short-term memory (LSTM), convolutional neural networks (CNN), and artificial neural networks (ANN). These models generally perform better when compared to traditional state-of-the-art methods in neural language, audio, visual processing, and numerous other sensor-based problems. The impact of deep learning on applications that use computer vision, such as object detection, activity recognition, motion tracking, and semantic segmentation, is profound [14].

A video is an arrangement of uninterrupted frames or images that creates a smooth and continuous impression for the individual viewing. The application of CNNs in the video field is broad. Examples of typical applications include video analysis and classification, an estimation of a human pose, and human detection. In problems associated with videos, temporal dimensions present an added complexity. The use of 3D CNNs can assist in finding an object’s motion in the video. In the 3D CNN, an array of frames constitutes the input, and a 3D filter is applied to the convolution. For this reason, the temporal and spatial details can be exploited in the same convolution.

An essential part of object detection involves ascertaining the class to which an object in the video or image belongs and then localizing its position by placing it inside a bounding box drawn around it [15]. To solve such challenges, deep learning has been very robust and successful. Examples of common frameworks for object detection include the restricted Boltzmann machine and deep belief networks, recurrent neural networks (RNNs), and CNNs. It can be mentioned that RNNs are mostly used in the processing of temporal signals.

The process of image fusion involves collecting all of the crucial details from numerous images and using them to create fewer images, or, in most cases, a single image. That one image is more accurate and informative when compared to any single source image. This is because the former contains all of the important details. Image fusion is not only performed to have fewer images and more data but also seeks to create images that are more suitable for machine and human perception and easier to comprehend [16,17,18]. Combining pertinent information from several images into one image in computer vision is known as multisensory image fusion [19]. The image that results from such fusion has more details than any of the images used to construct it [20].

This paper proposes a human fall detection system using multi-stream 3D CNNs with fusion. The major contribution of the system is that each stream corresponds to one of the four phases of a human fall (standing or walking, falling, fallen, and at rest). Each phase is a sequence of frames that have a certain orientation(s). Based on these features, sequences are clustered into four phases. Feeding phase-wise frames to the 3D CNNs is a new concept in human fall detection. There are other works where multi-stream CNNs were used but for different representations of frames rather than for different phases. Some previous works just supplied a fixed number of frames to the CNN, thereby ignoring the semantic information (phases of human fall) of the frames, producing a low accuracy of the systems.

The rest of the paper is organized as follows. Section 2 provides a comprehensive review of human fall detection systems. Section 3 describes the proposed system. Section 4 deals with the database used in the experiments, the experimental results, and the discussion. Finally, Section 5 concludes the paper and provides some future research directions.

## 2. Related Works

There are two types of human fall detection systems: using wearable sensors and not using wearable sensors (or machine-vision-based). In this section, we provide a literature review of human fall detection systems using these two types. In addition, we also briefly describe some state-of-the-art machine learning methods and deep-learning-based methods for fall detection.

### 2.1. Wearable-Sensors-Based Human Fall Detection Systems

Wearable sensors are commonly used in fall detection. These sensors include accelerometers, pressure sensors, gyroscopes, tilt switches, and magnetometers. Wearable systems for fall detection are usually data-set-dependent. The advantages of wearable sensors are that they are robust to different environments and measure body motion data directly [21]. The disadvantages include that they are difficult to wear all of the time and are intrusive. Due to the sensor being part of a person’s body, it is inconvenient to carry the sensor all day. The nature of the elderly makes it difficult to wear continuously, and its battery life is limited. In addition, one of the observed drawbacks of wearable sensors is that the accuracy of fall classification and detection is impacted by the placement of the sensor.

A wrist wearable device for the fall detection system was proposed in [22]. Data were acquired using an accelerometer, magnetometer, and gyroscope. The system used machine learning methods and threshold methods. A computationally efficient system was proposed in [23]. In the system, machine learning was used to analyze the behavior of lower limb muscles to detect pre-falls. A wearable fall detection system was proposed that can be used in a smart household environment [24]. The system received data from the accelerometer and gyroscope. Wearable sensors (gyroscope, accelerometer, and compass) were attached at six positions on the subject’s body.

A fall detection based on start, impact, posture, and aftermath phases was proposed in [25]. The system generated alarms if the fall was critical, i.e., the subject was not able to recover after the fall. This discrimination of real falls from activities was carried out with the help of defined thresholds of acceleration and orientation that were learned with the SVM. A low-cost fall detection ambient assisted living system was proposed in [26] to assist elderly and handicapped people. It included vibration detector sensors for measuring footsteps, motion, and unusual activity. It made the falling decision in real time based on instant motion if it occurred in the environment.

The research conducted by [27] detects falls using a cloud technology platform of the Internet of Things (IoT) together with a convolutional neural network. The model is known as the CNN-3B3 Conv. However, images were not used when conducting the analysis. Instead, sensors were employed to play the role of the accelerometer of the end user, connected to a phone and wristwatch attached to the body. In measuring rotation velocity to detect falls, the authors in [28] used data gathered by accelerometer sensors. They accomplished this by applying machine learning models such as ensemble bagged tree (EBT), ANN, and k-NN. When sensor data are compared with video streaming, it can be noted that the former’s dimensionality is smaller, and their training and application can be swiftly accomplished.

### 2.2. Machine-Vision-Based Human Fall Detection Systems

Machine-vision-based systems do not require the wearing of any sensors, so they are non-intrusive. Machine-vision-based systems have been deployed in many applications, including human fall detection [29,30,31]. Two categories of ambient fall detection systems were proposed in [26]: active and passive. Their feasibility was evaluated based on five aspects: power connectivity, affordability, obtrusiveness, the environment of installation, and the complexity of installation. An ambient assisted living system based on a 3D range camera was proposed for detecting falls [32]. The fall was detected using time-of-flight technology, and elderly silhouette segmentation was performed on the data from a range of cameras that were not affected by shadows and illumination. The system was tested on a large dataset of fall events during activities of daily life and achieved satisfactory results.

Deep learning was used in detecting falls in range–Doppler radars, also known as frequency-modulated continuous wave radar [33]. Two domains were used in analyzing radar returns, namely the time–frequency domain (spectrogram) and the range domain. The fusion of these two domains’ features helped to enhance the performance of the classifier. The radar signal is attractive to use because of its non-obstructive illumination, non-intrusive sensing, and privacy preservation [34]. Doppler effects are when radar backscatters from humans in motion generate changes in the radar frequency. Radar performs a role in assisted living with its ability to detect, classify, and localize. The challenges associated with radar for fall detection are high false alarms, the presence of multiple persons in the radar field of view, and the occlusion of the fall due to large stationary items.

A fall detection system based on spatio-temporal context tracking over a 3D depth image captured by a Microsoft Kinect sensor was proposed in [35]. In this system, assuming only one elderly person was in the scene, the head position of the subject and the coefficients of the floor plane equation were obtained first using the single Gaussian model (SGM). A tracking algorithm tracked the human with respect to the floor. The experiments were performed in a simulated environment and a Kinect sensor was used for recording the real video sequence. A low-cost vision-based system insensitive to illumination and occlusion used to detect fall accidents leading to injuries was proposed in [36]. Three-dimensional information of human silhouettes was extracted using a single Microsoft Kinect sensor. An adaptive Gaussian mixture model was used to segment a human from the background.

The fall detection system in [37] relied on the Microsoft Kinect camera to extract RGB data and depth data. In real-time, their system read each frame from the Kinect camera. A ground event segmentation by subtracting the background dynamically was performed. To identify falls within a household environment, the authors in [38] used a Microsoft Kinect. This is a process that happens in two steps. The first step involves following an individual for some time and establishing a time-series sequence of movement information. The main reason behind this is to divide the RGB-D picture into vertical events. The second step involves using a collection of choice trees for forecasting a potential fall depending on the sequential data.

Authors in [39] identified older adults falling using 3D depth images obtained using a Microsoft Kinect sensor. The target and background processing steps were accomplished with a median filter. Employing a method that subtracts the frames in the background from the picture, it is possible to see the silhouette of the individual traveling through a distance in the images. Using depth pictures to generate a disparity map requires using vertical and horizontal projection histogram data. The process for the identification of falls using a 2D CNN is discussed in [40]. The system recognizes activity from photos obtained using Microsoft Kinect. The new data collected by the researchers highlight indoor conditions in the development of a new dataset. To detect if a fall has happened, this method makes use of background elimination on the RGB-D, 2D CNN-provided posture classification, and depth information.

### 2.3. Machine-Learning-Based Human Fall Detection Systems

The paper in [41] discusses a vision-based technique used to detect a fall accident. The technique used in this research was based on the temporal slowness principle. Slow feature analysis (SFA) used high-level semantic contents and compared them with the posture of the fall incident (six shape features) extracted from the covered silhouette. In a system described in [42], features were generated using a DeeperCut model. The model extracted the human skeleton and decomposed it into five parts—two arms, two legs, and one head—which were used to generate 14 data points. The features were fed into a deep neural network, which had an input layer, five fully connected layers that used arctan as the activation function, one output layer, and two other category softmax classifiers.

A computer-vision-based fall detection system was proposed in [43]. This was different from other works that relied on non-vision approaches, such as those that used accelerometers. Considering the past challenges, such as the use of RGB images and high computational complexity, this work focused on creating a depth-camera-based vision approach, which is much faster, more accurate, and very robust. Fall detection was performed utilizing the SVM, trained with free fall body simulations, and coupled with a post-fall recovery analysis to help reduce false positives. The system reported no false positives, and only several falls were undetected.

A multistage computer-vision-based fall detection system was proposed in [44]. First, a video sequence was transformed into image frames with human subjects being extracted through ViBe and image post-processing with bounding box detection. Then, the first stage extracted features from these images using PCANet. This was carried out through a process of patch-mean removal and a principal component analysis (PCA) filter over an image. In the second stage, the features were fed into the SVM to classify the image as one of three categories: falling, fallen, or standing. Now, with a sequence of classified images, the output was fed into a second SVM that classified whether an individual had fallen or not. In the system in [45], the authors first performed background subtraction using a Gaussian distribution. Each pixel was modeled using Gaussian distribution with updated parameters to handle variations in illumination (slow or sudden) and the placement or removal of static objects. With the highlighted blobs of pixels, they could identify the foreground objects. These foreground objects were further smoothed by post-processing the image frame to remove isolated pixels and fill holes detected in the foreground. Points of interest were sampled throughout the image and matched to blobs.

Several researchers have focused on addressing the challenge of fall detection. For instance, [46] proposed a fall detection strategy based on the scene’s characteristics, including the human aspect ratio, the central rate of change in numerous frames, and the effective area ratio of the body. The main emphasis of the researchers in [46] was the improvement in accuracy while also attempting to reduce the probability of incorrect classifications. An idea for fall detection based on images that could be effective in nursing homes was presented by [47]. The researcher focused on accidents related to sitting on a chair and standing. The technique was based on first locating the body in the picture using an object detector. The location of the body in the image was evaluated in connection with the chair in the immediate area. Using the vertical and horizontal distance between the chair and the individual, the algorithm could determine the risk of falling. Their method relied on one picture when identifying falls, as it did not measure speed by recording the body’s dynamics or movement.

Four cameras at numerous angles and settings were used by [48] to generate a dataset for training a model. Using a median filter, the model developed removed the background before performing morphological techniques to divide the area in which an individual is located. Falls were identified by combining the segmented information with four features. These included the velocity of the head’s movement, the center of gravity’s speed of movement, and the proportion of the individual to the picture’s size. The attributes obtained were inputted into an SVM classifier to guess the potential for a fall. The SVM model formed the basis of this study and is responsible for the production of the most optimal global separation between the classes [49]. This leads to restrictions concerning generalization for new samples with an inadequate description of features in the trained dataset [50]. The employment of the media filter made it possible for [51] to efficiently get rid of the backdrop, resulting in some improvements.

### 2.4. Deep-Learning-Based Human Fall Detection Systems

A system based on a 3D CNN and LSTM was developed to improve the performance of existing methods, such as wearable-sensor-based and ambient-sensor-based methods [52]. It was found that videos captured by multiple cameras performed better compared to videos captured by a single kinematic camera. In addition, an LSTM-based mechanism was employed in combination with a 3D CNN to locate the activity in the image. The authors in [53] used a time–frequency (TF) analysis for fall detection because the TF analysis can extract high-order velocity components of different parts of the human body under motion. A CNN and a gated recurrent network were applied followed by a softmax regression classifier in the system.

A system for human fall detection on furniture based on deep learning and activity features was realized to reduce the existing limitations of other systems, such as auxiliary-equipment-based systems and vision-based systems [54]. This system first performed scene analysis using a deep learning method called faster R-CNN to detect human location and furniture, such as a sofa. The relationship between humans and furniture was detected in scene analysis. The authors in [55] proposed a system that used a deep learning approach that could work in single-camera or multi-camera situations. Using an improved version of ViBe (called IViBe), they subtracted the background to obtain a silhouette of a person. This was used to create a motion history image (MHI) and a dynamic image (DI). These images were fed into the three streams of the CNN. The three streams correspond to MHI, DI, and human silhouette sequences. Though their approach was computationally heavy, it illustrated a greater balance between sensitivity and specificity, beating out several other state-of-the-art models on the same dataset.

A CNN-based fall detection system was proposed in [56] by stating the motivations of independent environmental features, minimization of hand-engineering features, and higher levels of generalization. To achieve the minimization of hand-engineered features, they used CNNs by using a three-step training process. In the system in [57], the Deep ConvNet was trained on phases of a fall incident. To train the network, the frames of a training video were merged into a single image to make the analysis easy, and the dynamic image technique was used. For detecting a fall incident, a sliding window approach was used to find the correct sequence of the specific four actions.

Fall detection using low-resolution thermal sensors and recurrent neural networks (RNNs) was proposed in [58]. The sensors were placed in a pair; the first pair was placed at 10 cm whereas the second pair was placed at 1 m above the ground. The deep learning architecture had four layers: a 1D convolution layer with a ReLU activation function, max pooling layer (two-pool size), RNN layers, and dense layer (Sigmoid) that outputs 0 or 1 based on Fall and no Fall.

It is posited by [59] that people do not frequently fall off objects. For this reason, it is challenging to employ a supervised classification algorithm when assessing the potential that someone may fall from something. To frame the fall detection issue as an anomaly detection problem, a semi-supervised learning-based strategy was used. Deep spatiotemporal convolutional autoencoders were employed for understanding the temporal and spatial characteristics of unusual activity using non-invasive detection. The researchers conducted tests and studies on depth and thermal cameras, together with non-invasive sensing modalities. When dealing with falls, the use of autoencoders when handling falls as an abnormality is perceived as an advantage in this research. This model includes a construction phase connected with autoencoders, resulting in a boosted computation ability. This presents one of the drawbacks of the model. In addition, the optimal metrics can be obtained by using pictures obtained from depth cameras. This has led to this apparatus being progressively relied on.

A new dataset was introduced in [60]. This dataset consists of several categories of activities, including falls. The same scholars made available an algorithm that can be used in amalgamating the data gathered from the two channels based on the study of [61] and employed an RCNN [62]. RGB photos are transmitted into the first channel, an inception 3D (I3D) proposed by [63]. The second channel is an ICD and uses optical flow-processed identical images. With regard to the benefits of the work by [60], it is vital to highlight the creation of the novel dataset with keyframe marks and the amalgamation of several channels, which are broadly common in research involving action recognition but represent a creative method when it comes to fall detection. It can also be noted that this study embodies a novel approach toward the challenge of detecting falls. Numerous other categories matched many activities, and the sample quality was relatively uneven regarding falling, representing a limitation of the dataset. A huge proportion of the images were obtained from movies, where the dynamics of the fall were recreated digitally. In comparison to real photographs gathered from security cameras, the use of specific filters led to a loss of the naturalness of the situation.

Based on the literature review, we found that deep learning has recently been used for the system. Many types of features are also used. The missing ingredients are the use of multi-stream CNNs for different phases of the fall, and an efficient fusion network to fuse features from different phases. The only existing multi-stream CNNs were used for different image representations [55] rather than for different phases of the fall.

## 3. Materials and Methods

In this section, we present an overview of the proposed human fall detection method. This is followed by a description of the dataset used for the proposed method experiments and evaluations. After that, we illustrate our approach in preprocessing video sequences. Finally, we describe, in detail, the proposed classification model that identifies instances of human fall within a given video sequence.

### 3.1. Proposed Method

Since deep learning has achieved very good results in many areas of image and video processing, and it is still an interesting research area for human fall detection and identification, our goal in this proposed study was the development of a vision-based human fall detection method that utilizes multi-streams of 3D CNNs. We chose a video-based approach because wearable sensors are difficult to manage by an old person.

**Figure 1:**
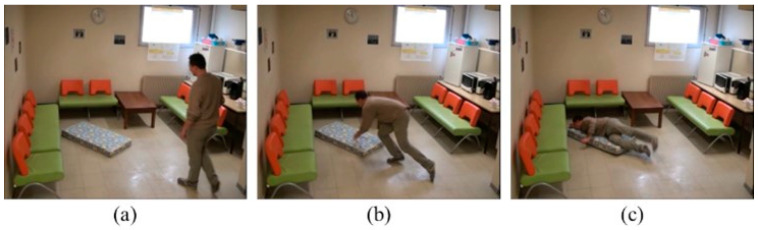
Human fall incident: (**a**) standing, (**b**) falling, (**c**) fallen.

An event of a human fall incident happens within a live or video scene that is translated into a series of images (video sequences). Such a scene usually consists of sequences of standing (or sitting), falling from an upper level to a lower level, such as ground, and being at rest. Figure 1 shows an example of the human fall sequence of standing, falling, and fallen.

Daily life activities in surveillance videos are diversified, and wide-scale event features can be generated by absorbing the appearance and motion information of the target person. Training a video-level CNN model can fulfill this requirement.

Two-dimensional convolution nets only extract the features at the frame or image level and ignores the dynamics of the object over a batch of frames [64,65]. The 3D CNN, in contrast, has the ability to extract the features from the spatial and temporal dimensions [66]. Thus, it is suitable for video data processing. In addition, multi-stream CNNs are a new trend used to deal with video data and have been used in several video-based classification systems [55,67,68].

**Figure 2:**
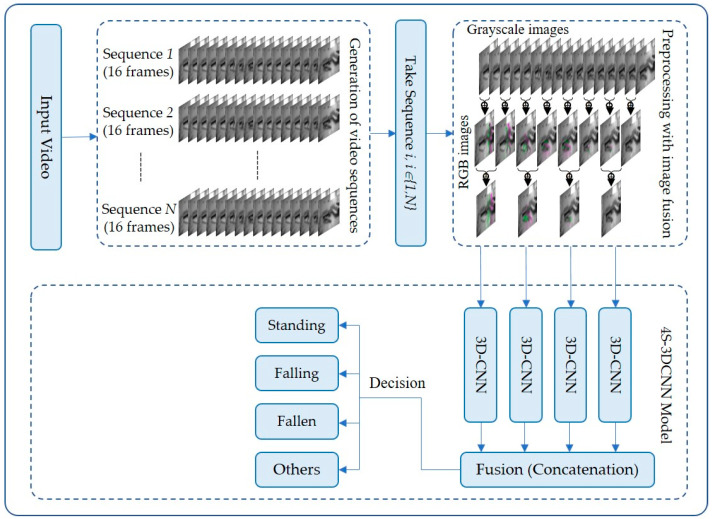
A simplified block diagram of the proposed fall detection system.

**Figure 3:**
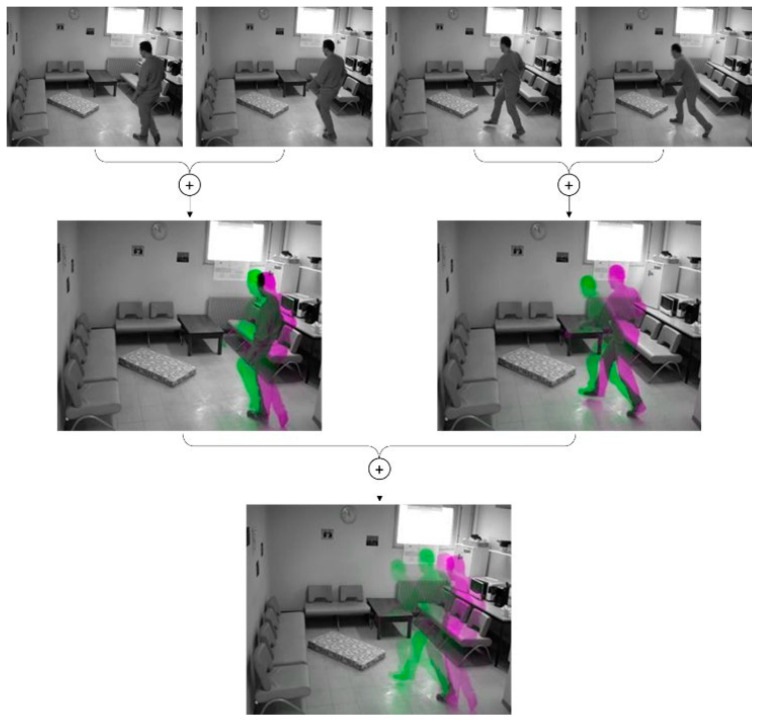
Two-level image fusion of 4 consecutive video frames.

Given the above discussion, the proposed work aimed to develop a vision-based human fall detection method using a multi-stream 3D CNN model based on a 4-stream (or branch) architecture (4S-3DCNN). The method starts by reading 16 frames from an input video, which represents approximately half of a second of a live video, given the video is recorded at 30 frames per second (fps). Then, it converts those frames into gray-scale images. After that, the method applies an image fusion technique to the input frames as a preprocessing step that results in four RGB preprocessed images. The preprocessed images highlight the spatial and temporal information within the 16 input frames. Figure 2 shows an overall block diagram of the proposed fall detection system. Figure 3 shows an example of how the fusion of consecutive grayscale images of human movement produces an RGB image that colorizes the temporal differences between fused images. Section 3.2 describes the utilized preprocessing approach in detail. Following that, the four preprocessed images are inputted to the proposed multi-stream 3D CNN model that uses a 4-branch architecture, wherein each branch deals with one image to learn features at different but consecutive spatial and temporal information. Section 3.3 illustrates the proposed multi-stream 3D CNN model architecture and specifications in detail.

### 3.2. Pre-Processing of Video Sequences

The undertaken preprocessing approach in the proposed work was based on the image fusion technique, which is based on combining relevant information from two or more images into a single image [69,70]. In our case, fusing two temporal frames of an input video sequence highlights the movement features, which were used for scene understanding and classification [71].

By having 16 frames from an input video sequence that were converted to grayscale and resized to 128 × 128 in height and width dimensions, the goal was to obtain four fused images to be used as input to the proposed multi-stream 3D CNN model. Within the proposed model that uses a 4-branch architecture, each image is handled by one branch.

**Figure 4:**
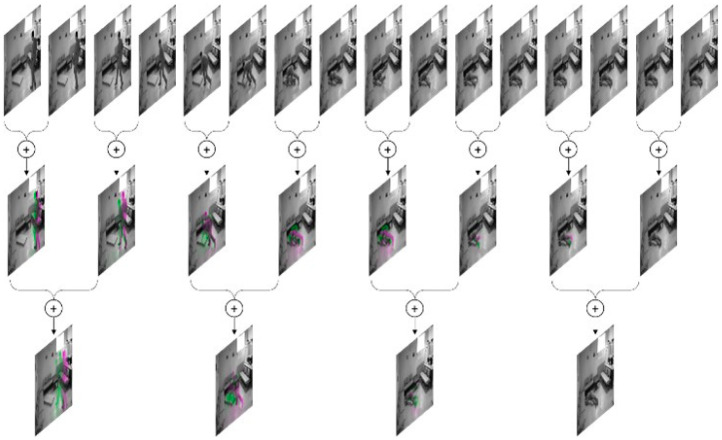
Two-level image fusion to produce 4 fused images out of the 16 input frames.

To produce four fused images from 16 input frames, the fusion process uses two levels of image fusion. At the first level, every odd frame with the one following is fused, resulting in eight fused frames. Following that, the second level of the same fusion approach is applied, which produces four preprocessed images by fusion. To better illustrate the conducted process of two-level fusion, Figure 3 shows an example of how image fusion of four consecutive video frames can clearly highlight movement features. However, Figure 4 shows the approach to producing four fused images out of the 16 input frames, each representing the two-level fusion of every 4 consecutive frames of the input video sequence.

The two-level fusion can be formulated as follows. *F_i_* corresponds to *i*-th frame.

First-level fusion:

$$A_{1}=fuse(F_{1}+F_{2});\;\;A_{2}=fuse(F_{3}+F_{4});\ldots .A_{8}=fuse(F_{15}+F_{16})$$

Second-level fusion:

$$B_{1}=fuse(A_{1}+A_{2});\;B_{2}=fuse(A_{3}+A_{4});\;B_{3}=fuse(A_{5}+A_{6});\;B_{4}=fuse(A_{7}+A_{8});\;\;$$

### 3.3. Model Architecture and Specifications

**Figure 5:**
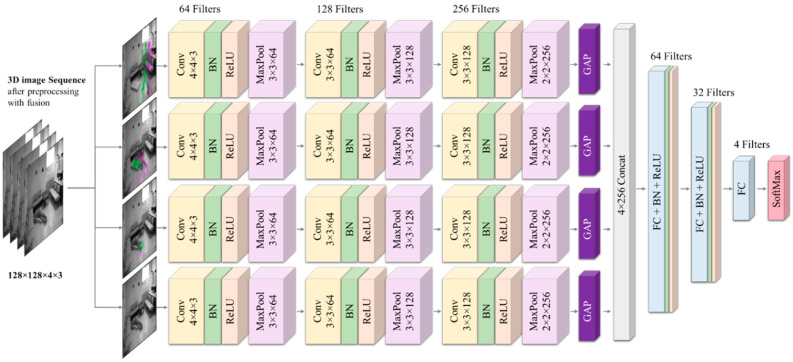
The proposed 4S-3DCNN model architecture for human fall detection.

The proposed multi-stream 3D CNN model was based on a 4-branch architecture with 67 layers as shown in Figure 5. There are 12 3D-convolutional (conv) blocks, with each block starting with a 3-DConv layer, after which, there are batch normalization (BN) and rectified linear unit (ReLU) layers, respectively. These layers are used complementarily in the model within each 3-DConv block, in which, the nonlinearity is introduced into the model by the ReLU layer by obtaining the targeted pixels from the BN layer. A thresholding operation is applied within the ReLU layer to retain the positive pixels and set the negative pixels to 0. In the proposed model, a max-pooling layer was positioned after each 3-DConv block, resulting in a total of 12 max-pooling layers. The significance of such pooling in the model is the ability to select only one pixel with the highest value compared to the other pixels of the pooling receptive field. This process allows for the extraction of relevant features and reduces the input size.

The input layer of this model receives a 3D image input of size 128 ×128 × 4× 3 that represents an input video sequence. The input size of this layer corresponds to the height, width, depth, and number of channels, respectively. The height and width are of size 128 because all input video frames are resized to this size in the preprocessing step. The depth of this layer represents the 4 fused images resulting from the preprocessing with each image having 3 channels that represent the number of channels. It is then passed to 4 branches using 4 splitting layers in which each split takes one of the 4 preprocessed images and passes it to one of the 4 branches of this model. The purpose of the 4 branches is to extract and learn features from the preprocessed images at 4 consecutive sequences that have different spatial and temporal information. Three consecutive Conv blocks are located on every branch, in which, the first Conv layer of each branch contains 64 filters with a size of 4 × 4 × 3 and stride of 2 ×2 × 3. This down-samples the branch input from 128 × 128 × 1 × 3 to 63 × 63 × 1 × 64. The second Conv layer of each branch includes 128 filters with a size of 3 × 3 × 64 and stride of 2 ×2 × 64, whereas the third Conv contains 256 filters with a size of 3 × 3 × 128 and stride of 1 × 1× 128. The depth of each filter size along with its stride corresponds to the number of filters used in its previous layer. The reason for this is to reduce the number of learnable parameters as well as the model’s computational cost.

For the 12 used pooling layers, each Conv block within each branch is followed by a max-pooling layer. The height and width of receptive field for the max-pooling layers are either 3 × 3 or 2 × 2 depending on whether the previous output feature map is odd or even, whereas the depth corresponds to the number of filters used in its previous layer.

A global average pooling (GAP) layer at the end of each branch is prime for the generation of channel descriptors for each branch. The generated GAP descriptors are fused using the concatenation layer, which is used as an input to two fully connected (FC) blocks. Each FC block includes an FC layer that has 64 and 32 filters for the first and second, respectively, after which, there are BN and ReLU layers.

At the end of this proposed model, an FC layer that has 4 neurons followed by a softmax was used as a classifier. The number of neurons in the final FC layer is based on the number of classes of interest, including standing, falling, fallen, and others.

The hyperparameters of the model, such as the number of filters, the size of filters, the stride, the number of neurons in the FC layers, and the total number of convolutional layers, were chosen empirically, keeping an eye on the model complexity. The number of filters in successive convolutional layers was doubled, whereas the number of neurons in the successive FC layers was halved (expand and shrink approach). The size of filters, the stride, and the max-pooling were chosen is such a way that the final image size before the GAP became 4 × 4, which is a meaningful size of an image.

Table 1 shows the architecture of the proposed model.

## 4. Experiments

### 4.1. Database Description

In the proposed work, we considered the Le2i fall detection dataset [72]. This dataset comprised 192 fall detection videos, as well as 57 normal videos. The latter contains activities of daily living, such as walking, sitting down, standing up, and squatting. Based on a fixed camera, these were obtained at four different locations (home, coffee shop, office, and lecture room) by actors. The actors wore a variety of clothing and attempted to simulate various kinds of normal daily activities and falls to increase the diversity of the dataset. Furthermore, this dataset presents occlusions, shadows, and variations in illumination, which were manually annotated. The beginning and ending frames of each fall were manually annotated.

### 4.2. Dataset Preparation

For developing each CNN model described in the proposed method section, the training and validation datasets were prepared manually by selecting a controlled number of videos from the Le2i fall detection dataset. Almost every video of this dataset includes a human fall action that consists of consecutive actions of standing, falling, and fallen, as well as other actions prior to the standing, such as sitting. From such videos, we cut the range of these actions in separated videos to consider them as classes for training and validation. Following that, every video within each class was separated into video sequences of 16 frames, each to be used as an input to the preprocessing step as described in Section 3.2. Although videos of each class may differ in the number of frames, ranging between 63 to 144 frames on average, we applied an overlapping approach to generate a reasonable number of video sequences, each having 16 frames. Within the overlapping approach, we excluded any extra resulting frames that were less than 16 frames. Equation (1) states the formula for generating overlapping video sequences from an input video clip. We considered an overlapping factor of 5 in generating video sequences.

(1) $$Num\;of\;Sequences=floor\left(\frac{Num\;of\;Video\;Frames-16}{Overlapping\;Factor}+1\right)$$

Given this, the total number of generated videos for each action is 455. However, the total number of generated video sequences from all classes is 6392. Table 2 provides details about the generated videos for each class with the generated sequences for the class.

### 4.3. Experiments and Evaluations

The machine used for the experiments was an Intel^®^ Core™ i9-9900K CPU @ 3.60 GHz and 64 GB of RAM running a 64-bit windows 10 operating system. It was also equipped with an NVIDIA GeForce RTX 2080 Ti with 11 GB of GPU memory. The suitability of the machine in experimenting was determined by running a 64-bit version of MATLAB R2022b.

It was observed that there was a need to increase the generalization and reliability of the results; however, this was not possible due to hardware limitations. Therefore, the proposed model validates the models using a three-fold cross-validation method by training and testing the models three times, where one fold was used for testing, and the remaining two were used for training and validation. For the training and validation, around 85% of the data were used for training, and the rest for validation.

The experiments conducted in this work included a classification performance for the fine-tuned pre-trained models (i.e., GoogleNet, SqueezeNet, ResNet18, and DarkNet19) and the proposed multi-stream 3D CNN model. Using such pre-trained models facilitates a fair comparison with the proposed multi-stream 3D CNN model. By applying the three-fold cross-validation technique to every model within the conducted experiments, each fold has 2130 3D image sequences that were used for testing, whereas the remaining 3836 and 426 sequences were used for training and validation, respectively. The Adam optimization algorithm is considered to be one of the most efficient among all available optimization methods since it outperforms its counterparts. Therefore, all trained models were also trained by the Adam optimization algorithm with a gradient decay factor of 0. The initial learning rate was set at 0.001, while the regularization factor deemed most appropriate was 0.0001. Due to the limitations of the memory, the models were trained for 100 epochs with a minibatch size of 8.

The test evaluation is based on accuracy, sensitivity, specificity, and precision, which are the most commonly used performance indicators. Through three-fold cross-validation, the mean and standard deviation of the measurements were reported over three testing folds. As shown in Table 3, performance measurements were derived using the concepts of true positive (TP), true negative (TN), false positive (FP), and false negative (FN).

Evaluating our proposed method for human fall detection was performed using a three-fold cross-validation approach to ensure the generalization and reliability of the results. As mentioned in Section 3.2, every input video sequence was preprocessed by fusion to produce four fused images from 16 frames, after being converted to grayscale and resized to 128 × 128 in the height and width dimensions. These four fused images were used as the input to every evaluated model.

**Figure 6:**
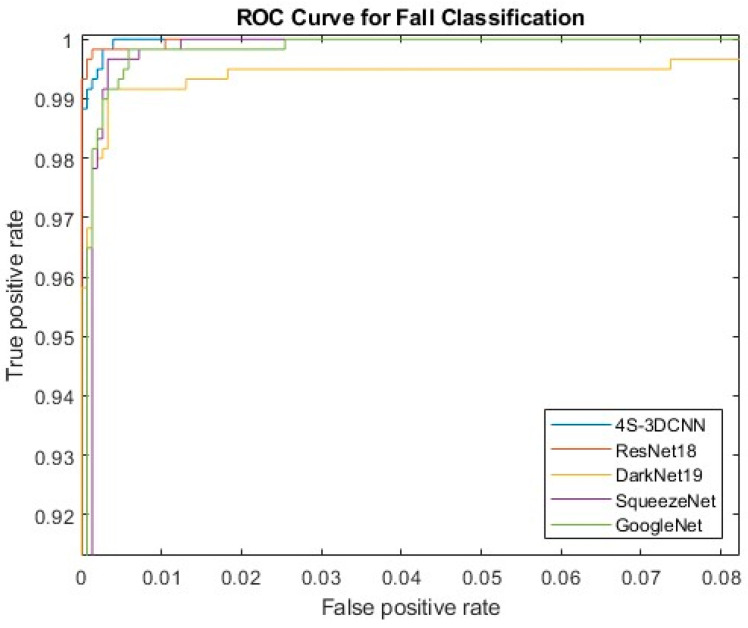
ROC curves for each trained model.

The results of experimenting with three-fold cross-validation for each of the fine-tuned pre-trained CNN models (i.e., GoogleNet, SqueezeNet, ResNet18, and DarkNet19) in addition to the proposed multi-stream 3D CNN model are shown in Table 4. These results are reported by averaging the resulting (test set) metrics with the standard deviation (std) of each metric of the three crossed validated folds. The proposed multi-stream 3D CNN model archived the best results among others compared to the state-of-the-art models, having an average accuracy, sensitivity, specificity, and precision of 99.03%, 99.00%, 99.68%, and 99.00%, respectively. In addition, the standard deviations of these metrics were 0.44%, 0.46%, 0.15%, and 0.46%, respectively. This proves the potential of the proposed multi-stream 3D CNN model to improve the detection of human falls. In addition, Figure 6 shows the ROC curves, which indicate that the proposed model outperforms other state-of-the-art models.

Information density is a measure of the performance of the models concerning how the accuracy behaves with the number of hyperparameters. It is defined by the accuracy (in percentage) obtained by the model divided by the number of hyperparameters in millions (M). A good model has a high information density. Table 5 shows the accuracy, number of hyperparameters, number of layers, and information density of the models. From the table, we can see that the information density of the proposed model is the highest by a distance. The proposed model is light, yet achieves high accuracy.

### 4.4. Discussion

The proposed method aims to detect human falls using a vision-based multi-stream 3D CNN model with the fusion of video frame sequences to capture consecutive spatial and temporal information. Given the results in Table 4, it can be seen that GoogleNet, SqueezeNet, and DarkNet19 performed similarly, resulting in a mean accuracy of 97.06%, 97.62%, and 97.45%, with a standard deviation of 1.09%, 0.29%, and 0.12%, respectively. Likewise, for the other metrics, these models had a sensitivity of 97.45%, 97.57%, and 97.35%, specificity of 99.19%, 99.22%, and 99.15%, and precision of 97.51%, 97.55%, and 97.39%, respectively. Although the SqueezeNet slightly outperformed both of GoogleNet and DarkNet19 in all metrics, with a difference of 0.56% and 0.17% in accuracy, 0.12% and 0.22% in sensitivity, 0.38% and 0.07% in specificity, and 0.04% and 0.16% in precision, respectively, the DarkNet19 standard deviations across the three cross-validated folds were better in performance.

ResNet18 was associated with a better performance, with a mean accuracy, sensitivity, specificity, and precision of 98.75%, 98.73%, 99.59%, and 98.71%, with standard deviations of 0.09%, 0.08%, 0.03%, and 0.09%, respectively. The proposed 4S-3DCNN model outperformed the other fine-tuned state-of-the-art models on every metric, with values of 99.03%, 99.00%, 99.68%, and 99.00% for accuracy, sensitivity, specificity, and precision, respectively. Although its standard deviations did not outperform other models, except for GoogleNet, a difference of 0.28% in mean accuracy, 0.27% in mean sensitivity, 0.09% in mean specificity, and 0.29% in mean precision was achieved over ResNet18. This indicates the effectiveness of our proposed method in comparison with four state-of-the-art fine-tuned models (i.e., GoogleNet, SqueezeNet, ResNet18, and DarkNet19).

On the other hand, a comparison with similar works is given in Table 6. These results are compared to the results from each author experimenting with their proposed method on the Le2i dataset. This comparison includes four non-CNN-based methods and four CNN-based methods, along with our proposed method.

The work of Chamle et al. [73] utilized the background subtraction approach to detect and mark movement between video frames with a rectangular and elliptical bounding box. Following that, the gradient boosting classifier was used to classify the fall by extracting the features of the aspect ratio, fall angle, and silhouette height from the marked bounding box. Their proposed method, without mentioning the number of training, testing, and validation sets, resulted in an accuracy of 79.30%, sensitivity of 84.30%, specificity of 73.07%, and precision of 79.40%. Alaoui et al. [74] computed human silhouettes using optical flow to produce motion vectors for their fall detection by applying a directional distribution method called von Mises distribution. They achieved an accuracy of 90.90%, sensitivity of 94.56%, specificity of 81.08%, and precision of 90.84%. Poonsri et al. [75] used principal component analysis (PCA) to extract aspect and area ratios, as well as the orientation from the human silhouette, to determine fall events. Their results achieved 86.21%, 93.18%, 64.29%, and 91.11% in accuracy, sensitivity, specificity, and precision, respectively. In the reference [76], the authors proposed a fall detection method based on human body skeleton features with the computing of similarity scores between sequences using the dynamic time warping (DTW) algorithm. In their classification between fall and non-fall sequences using the leave-one-out protocol, an SVM classifier with a linear kernel was used, which resulted in an accuracy, sensitivity, specificity, and precision of 93.67%, 100.00%, 87.00%, and 83.62%, respectively.

Núñez et al. [56] introduced a CNN-based fall detection method that included pre-training on the ImageNet dataset, tuning on an action-motion dataset (UCF101 dataset), and a final fine-tuning for the fall detection task. Their method was based on constructing an optical flow block of each 10 consecutive frames and using them as an input to their method. Their best-reported results, based on lighting manipulations to training sets, achieved a 97.00% accuracy, 99.00% sensitivity, and 97.00% specificity, with no score of precision reported. Zou et al. [77] presented a 3D-CNN-based method for the detection of human fall incidents. Using a sequence of 16 frames, 3D features were extracted using a 3D-CNN model followed by the tube anchors generation layer and a softmax classification layer. Their results on the Le2i dataset achieved a 97.23% accuracy, 100.00% sensitivity, and 97.04% specificity. Vishnu et al. [78] proposed a fall motion mixture model (FMMM) constructed from a Gaussian mixture model (GMM) to detect fall and non-fall events. In their feature extraction approach, they adopted a histogram of optical flow (HOF) and motion boundary histogram (MBH) to form a fall motion vector from 15 consecutive frames. Their experiments achieved an accuracy of 78.50%, sensitivity of 93.00%, and precision of 81.50%, without reporting the specificity.

Our proposed method based on a multi-stream 3D CNN with a four-branch architecture (4S-3DCNN) greatly outperforms similar works in accuracy, specificity, and precision. Although the works of Zou et al. [77] and Youssfi et al. [76] show a better sensitivity with only a difference of 1% compared to our method, Zou et al. [77] did not consider any type of cross-validation in their evaluation, while Youssfi et al. [76] applied only the leave-one-out cross-validation protocol. Given that our evaluation applies the three-fold cross-validation approach, this proves the generalization favorability of our 4S-3DCNN model, with its low susceptibility to overfitting.

**Table 6:** Comparison of the proposed 4S-3DCNN with similar works on the Le2i dataset.

Model	Accuracy	Sensitivity	Specificity	Precision
Chamle et al. [73]	79.30%	84.30%	73.07%	79.40%
Núñez et al. [56]	97.00%	99.00%	97.00%	-
Alaoui, et al. [74]	90.90%	94.56%	81.08%	90.84%
Poonsri et al. [75]	86.21%	93.18%	64.29%	91.11%
Zou et al. [77]	97.23%	100.00%	97.04%	-
Vishnu et al. [78]	78.50%	93.00%	-	81.50%
Youssfi et al. [76]	93.67%	100.00%	87.00%	83.62%
Proposed 4S-3DCNN	99.03%	99.00%	99.68%	99.00%

## 5. Conclusions

This paper proposes an automatic vision-based human fall detection method using a multi-stream 3D CNN that is based on a four-branch architecture (4S-3DCNN). A preprocessing step was undertaken to utilize the image fusion method in two levels of fusion to produce four consecutive fused images out of 16 input video frames. Each branch of the proposed 4S-3DCNN deals with one of the four preprocessed images to extract and learn features at different but consecutive spatial and temporal information. The evaluation included the use of more than 6392 generated sequences, 16 frames per each, from the publicly available Le2i fall detection dataset. Using a three-fold cross-validation technique, the model outperformed four state-of-the-art deep learning models (i.e., GoogleNet, SqueezeNet, ResNet18, and DarkNet19). The proposed method achieved an averaged accuracy, sensitivity, specificity, and precision of 99.03%, 99.00%, 99.68%, and 99.00%, respectively. This proves that the proposed multi-stream 3D CNN with the two-level image fusion approach can assist in human fall incident detection.

Nevertheless, the proposed method is limited to the detection of human falls within a scene that only has one person without any segmentation or localization of the presented human. In addition, the conducted experiments were only performed on the Le2i fall detection dataset. In future work, we intend to investigate the performance of applying human segmentation approaches to only segment the fallen human without any unrelated spatial or temporal information. In addition, different publicly available datasets will be investigated and used to better evaluate the proposed method.

## Figures and Tables

**Table 1:** The architecture of the proposed 4S-3DCNN.

Layer Type	Number of Filters	Size of Kernel	Size of Stride	Size of Feature Map
Input Layer	128 × 128 × 4 × 3			
1st Convolutional layer				
Branch 1	64	4 × 4 × 3	2 ×2 × 3	63 × 63 × 1 × 64
Branch 2	64	4 × 4 × 3	2 × 2 × 3	63 × 63 × 1 × 64
Branch 3	64	4 × 4 × 3	2 × 2 × 3	63 × 63 × 1 × 64
Branch 4	64	4 × 4 × 3	2 × 2 × 3	63 × 63 × 1 × 64
2nd Convolutional layer				
Branch 1	128	3 × 3 × 64	2 × 2 × 64	15 × 15 × 1 × 128
Branch 2	128	3 × 3 × 64	2 × 2 × 64	15 × 15 × 1 × 128
Branch 3	128	3 × 3 × 64	2 × 2 × 64	15 × 15 × 1 × 128
Branch 4	128	3 × 3 × 64	2 × 2 × 64	15 × 15 × 1 × 128
3rd Convolutional layer				
Branch 1	256	3 × 3 × 128	1 × 1 × 128	5 × 5 × 1 × 256
Branch 2	256	3 × 3 × 128	1 × 1 × 128	5 × 5 × 1 × 256
Branch 3	256	3 × 3 × 128	1 × 1 × 128	5 × 5 × 1 × 256
Branch 4	256	3 × 3 × 128	1 × 1 × 128	5 × 5 × 1 × 256
1st Max Pooling layer				
Branch 1		3 × 3 × 1	2 × 2 × 64	31 × 31 × 1 × 64
Branch 2		3 × 3 × 1	2 × 2 × 64	31 × 31 × 1 × 64
Branch 3		3 × 3 × 1	2 × 2 × 64	31 × 31 × 1 × 64
Branch 4		3 × 3 × 1	2 × 2 × 64	31 × 31 × 1 × 64
2nd Max Pooling layer				
Branch 1		3 × 3 × 1	2 × 2 × 128	7 × 7 × 1 × 128
Branch 2		3 × 3 × 1	2 × 2 × 128	7 × 7 × 1 × 128
Branch 3		3 × 3 × 1	2 × 2 × 128	7 × 7 × 1 × 128
Branch 4		3 × 3 × 1	2 × 2 × 128	7 × 7 × 1 × 128
3rd Max Pooling layer				
Branch 1		2 × 2 × 1	1 × 1 × 256	4 × 4 × 1 × 256
Branch 2		2 × 2 × 1	1 × 1 × 256	4 × 4 × 1 × 256
Branch 3		2 × 2 × 1	1 × 1 × 256	4 × 4 × 1 × 256
Branch 4		2 × 2 × 1	1 × 1 × 256	4 × 4 × 1 × 256
Fully connected (FC) layer				
1st FC layer	64			1 × 1 × 1 × 64
2nd FC layer	32			1 × 1 × 1 × 32
3rd FC layer	4			1 × 1 × 1 × 4

**Table 2:** Dataset preparation.

Class	Generated Videos	Generated Sequences
Standing	128	1498
Falling	104	1464
Fallen	103	1793
Other	120	1637

**Table 3:** Performance measurement metrics.

Measurement	Equation	Equation No.
Accuracy	$\frac{TP\;+\;TN}{TP\;+\;TN\;+\;FP\;+\;FN}\left(\times 100\%\right)$	(2)
Sensitivity	$\frac{FP\;+\;FN}{TP\;+\;TN+\;+\;FP\;+\;FN}\left(\times 100\%\right)$	(3)
Specificity	$\frac{TP}{TP\;+\;FN}\left(\times 100\%\right)$	(4)
Precision	$\frac{TN}{FP\;+\;TN}\left(\times 100\%\right)$	(5)

**Table 4:** Human fall detection results using the 3-fold cross-validation approach (averaged over 3 folds of each model (mean ± std)).

Model	Accuracy	Sensitivity	Specificity	Precision
GoogleNet	97.06 ± 1.09	97.45 ± 1.18	99.19 ± 0.36	97.51 ± 1.05
SqueezeNet	97.62 ± 0.29	97.57 ± 0.31	99.22 ± 0.10	97.55 ± 0.30
ResNet18	98.75 ± 0.09	98.73 ± 0.08	99.59 ± 0.03	98.71 ± 0.09
DarkNet19	97.45 ± 0.12	97.35 ± 0.13	99.15 ± 0.04	97.39 ± 0.14
4S-3DCNN	99.03 ± 0.44	99.00 ± 0.46	99.68 ± 0.15	99.00 ± 0.46

**Table 5:** Information density comparison along with accuracy, number of parameters, and layers of the proposed 4S-3DCNN model with fine-tuned models.

Model	Accuracy (%)	No. of Parameters	No. of Layers	Information Density
GoogleNet	97.06%	14.5 M	142	6.694
SqueezeNet	97.62%	2.6 M	68	37.546
ResNet18	98.75%	33.1 M	71	2.983
DarkNet19	97.45%	57.9 M	64	1.523
Proposed 4S-3DCNN	99.03%	1.5 M	67	66.020
